# The direct and indirect impact of the COVID-19 pandemic on the care of patients with pituitary disease: a cross sectional study

**DOI:** 10.1007/s11102-020-01106-3

**Published:** 2020-11-24

**Authors:** Anneke Graf, Hani J. Marcus, Stephanie E. Baldeweg

**Affiliations:** 1 Department of Endocrinology, University College London Hospitals NHS Foundation Trust, London, UK; 2 Division of Neurosurgery, National Hospital for Neurology and Neurosurgery, University College London Hospitals NHS Foundation Trust, London, UK; 3 Division of Medicine, University College London, London, UK

**Keywords:** Pituitary, COVID-19, Pandemic, Impact, Direct, Indirect

## Abstract

**Purpose:**

The coronavirus disease 2019 (COVID-19) pandemic is widely believed to have had a major impact on the care of patients with pituitary disease. The virus itself may directly result in death, and patients with adrenal insufficiency, often a part of hypopituitarism, are thought to represent a particularly susceptible subgroup. Moreover, even in patients that do not contract the virus, the diversion of resources by healthcare institutions to manage the virus may indirectly result in delays in their management. To this end, the aim of this study was to determine the direct and indirect impact of the COVID-19 pandemic on patients with pituitary disease.

**Methods:**

A cross-sectional study design was adopted, with all adult patients seen by our pituitary service in the year prior to the nationwide lockdown on March 23rd 2020 invited to participate in a telephone survey.

**Results:**

In all, 412 patients (412/586; 70.3%) participated in the survey. 66 patients (66/412; 16.0%) reported having suspected COVID-19 infection. Of the 10 patients in this group tested for COVID-19 infection, three received a positive test result. No deaths due to COVID-19 were identified. 267 patients (267/412; 64.8%) experienced a delay or change in the planned care for their pituitary disease, with 100 patients (100/412; 24.3%) perceiving an impact to their care.

**Conclusions:**

Whilst only a small percentage of patients had confirmed or suspected COVID-19 infection, over half were still indirectly impacted by the pandemic through a delay or change to their planned care.

## Background

The worldwide spread of the viral strain that has caused the coronavirus disease 2019 (COVID-19) pandemic, along with the unprecedented pressure subsequently placed on healthcare, has promoted concerns that patients with chronic medical conditions, including those with pituitary disease, have been adversely affected [1, 2].

The morbidity and mortality of acute COVID-19 infection is of particular concern in the potentially susceptible subgroup of patients with pituitary disease and secondary adrenal insufficiency. This group is generally at increased risk of infection [3]. Patients on replacement glucocorticoids with acute suspected or confirmed COVID- 19 infection are recommended to follow ‘sick day rules’, as the course of illness may be further complicated by development of an adrenal crisis triggered by an infection [4].

The first COVID-19 cases were detected in the United Kingdom in late January 2020, with the case numbers rising rapidly in March 2020 [5]. On the 23rd of March 2020, strict social distancing measures were introduced by the United Kingdom (UK) Government, requiring citizens to ‘stay at home’ (commonly referred to as a period of ‘lockdown’). To help manage the impact on the National Health Service (NHS), the provision of emergency medical services was prioritised.

Whilst some studies examine the effects of natural disasters on chronic illness, including hurricanes, earthquakes and tsunamis [6–9], little is known about the impact of the current global pandemic. The aim of our work was to identify the proportion of our patients with pituitary disease who have been directly impacted by contracting the COVID-19 virus, or indirectly affected due to a delay or changes in their planned care.

## Methods

We adopted a cross-sectional study design and the Strengthening the Reporting of Observational Studies in Epidemiology (STROBE) Statement in the preparation of this manuscript. The study was registered as a Service Evaluation study with the University College London Hospital NHS Foundation Trust (UCLH) Clinical Audit Committee, with verbal consent sought from patients participating in the study.

### Settings and participants

The study was conducted at UCLH, which acts as a regional referral centre for patients with pituitary disease. As per recent national review by the Getting it Right First Time Initiative (GIRFT), we are the largest pituitary centre in the UK. Using electronic healthcare records, we identified all adult patients with pituitary disease attending the endocrine clinic of the senior author (SEB) at our institution, in the year prior to the nationwide lockdown, between 23rd of March 2019 to 22nd March 2020.

### Variables and data sources

**Fig. 1:**
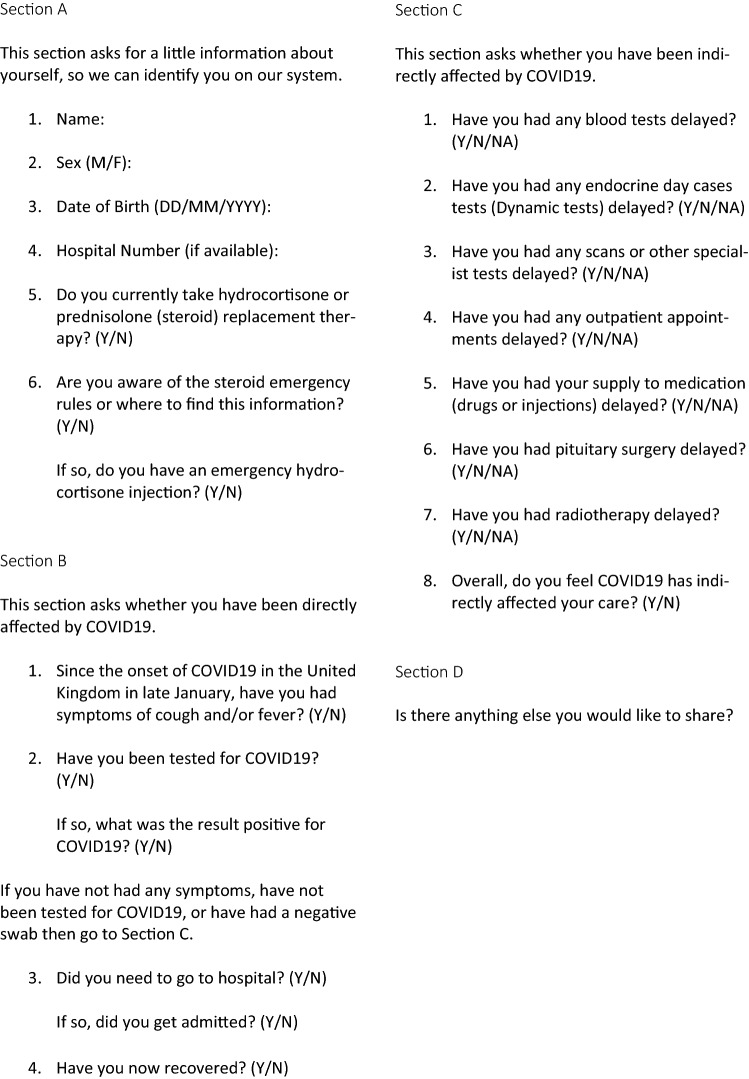
Patient survey

All patients had their electronic health records first reviewed to determine whether they were currently admitted or had died since their last clinical appointment, and in these cases a detailed case note review was performed to ascertain whether this was related to COVID-19. Otherwise, patients were surveyed by telephone between the 23rd May and 30th June 2020 (see Fig. 1), A second call was attempted on a different day if patients failed to answer the initial phone call. All surveys were conducted by the same doctor (AG). In each case patients were asked: firstly, their demographic details including age and sex; secondly, whether they were directly affected by COVID-19, including confirmed or suspected COVID-19 infection since the end of January 2020 and their awareness of sick-day rules; and thirdly, whether they were indirectly affected by COVID-19 following the introduction of lockdown measures in March 2020, including delays in their management. The interviewer (AG) cross checked any delays in management reported by the patients with the electronic data base record. At the conclusion of the survey we also asked whether they had anything else they would like to share.

### Study size and statistical methods

No formal power calculation was performed. Instead, the sample size was determined on a pragmatic approach in which we considered a minimum of 100 patients and a survey response rate of 50% sufficient for meaningful analysis, and we estimated that this would be achieved by including patients seen within the year prior to lockdown.

Data were recorded and analysed using Microsoft Excel. The mean and standard deviation were calculated for age.

## Results

In all, 586 patients met the inclusion criteria, with 412 (70.3%) completing the survey. The group surveyed had an age range from 18 to 88 years (mean = 48 years, standard deviation = 16 years) and consisted of 213 females (213/412; 51.7%). Of the 174 patients not surveyed; 167 were uncontactable by phone, three declined to take part, and two were unable to take part due to a language barrier. A further two patients were deceased, both of whom had died of causes unrelated to COVID-19.

### Direct impact

Sixty-six patients (66/412; 16.0%) had experienced symptoms consistent with COVID-19 infection [10] at any time since late January 2020, when the first COVID-19 cases were reported in the UK. Ten of these patients underwent a test for a current COVID-19 infection, with three receiving a positive result. Six patients presented to hospital due to their symptoms. One patient was admitted due to confirmed COVID-19 infection and has subsequently recovered and been discharged home.

One hundred and fifty-nine patients were taking steroid replacement therapy (159/412; 38.6%). Within this group, 154 patients (154/159; 96.9%) were aware of steroid emergency rules or where to find such information, and 113 (113/159; 71.1%) had an emergency intramuscular 100mg hydrocortisone injection available to them. Thirty patients taking steroid replacement therapy reported symptoms of COVID-19 infection (30/159, 18.9%), with two of the seven patients tested for COVID-19 infection in this group receiving a positive test result.

### Indirect impact

Overall, 267 of the patients (267/412; 64.8%) experienced a delay or change in the planned care for their pituitary disease (Table 1), and one hundred patients (100/412; 24.3%) reported that they felt the COVID-19 pandemic had indirectly affected their care as patients with pituitary disease.

**Table 1:** Types of delay or changes in planned care

Type of delay or change	Number of patients (%)
Delay in appointment	
Delay in outpatient appointment (endocrinology or neurosurgery)	65 (15.8%)
Planned face to face appointment conducted via telephone	134 (32.5%)
Delay in investigations	
Delay in pathology	200 (48.5%)
Delay in imaging	51 (12.4%) ^# *^ • MRI pituitary: 27 • DEXA scan: 25 • TTE: 1
Delay in endocrine dynamic test	7 (1.7%) ^##^ • ITT: 4 • OGTT: 2 • GST: 1
Delay in treatment	
Delay in pituitary radiotherapy	4 (1.0%)
Delay in pituitary surgery	2 (0.05%)
Difficulties obtaining prescribed endocrine medications	59 (14.3%) ^###^ • Testosterone or gonadotrophin 20 • Growth hormone:13 • Lanreotide: 4 • Hormone replacement therapy: 4 • Desmopressin: 2 • Cabergoline: 1 • Levothyroxine:1 • Denosumab: 1 • Prescription delays with local pharmacy or general practitioner: 15

^#^MRI: Magnetic resonance imaging; DEXA scan: dual-energy X-ray absorptiometry; TTE: transthoracic echocardiogram (for a patient on cabergoline)

*2 patients had both an MRI and a DEXA scan delayed

^##^ITT: insulin tolerance test; OGTT: oral glucose tolerance test; GST: glucagon stimulation test

^###^1 patient had 3 medication issues

Sixty-five patients (65/412; 15.8%) had their endocrine or neurosurgery outpatient appointment delayed and 134 (134/412; 32.5%) had their planned face to face appointment conducted via telephone instead. Two hundred patients (200/412; 48.5%) had pathology delayed (for example, blood tests planned to assess or monitor pituitary hormone levels were not performed), 51 (51/412; 12.4%) had imaging delayed and seven (7/412; 1.7%) had a delay in a planned endocrine dynamic test (to assess the hormonal response after stimulation or suppression of a particular hormonal axis for example, an insulin tolerance test). Fifty-nine patients (59/412; 14.3%) experienced difficulties obtaining their prescribed endocrine medications, either in primary or secondary care. No patients reported difficulty obtaining their prescribed hydrocortisone replacement, although five experienced a delay in taking medication to suppress pituitary hormone overproduction (one cabergoline and four lanreotide). Four patients had a delay in pituitary radiotherapy treatment and two patients had planned pituitary surgery delayed.

### Other

A common theme in the free comment section related to the psychological impact of the pandemic. Eighteen patients reported low mood and anxiety related to the lockdown, the risk of contracting COVID-19 and the uncertainty of the future. Seventeen patients also reported delays in the care of medical conditions unrelated to their pituitary disease.

## Discussion and conclusions

### Principal findings

Only a small percentage of patients with a pituitary disorder at our institution reported having confirmed or suspected COVID-19 infection, but over half were indirectly impacted by the pandemic through a delay or change to their planned care. Nonetheless, the majority of impacted patients remained positive and perceived the impact on their care to be low. Moreover, almost all patients on long-term replacement steroids were knowledgeable of sick day management.

### Comparison with other studies

In our study, we found 66 patients (66/412; 16.0%) had experienced symptoms consistent with a COVID-19 infection, albeit with not all of these confirmed due to the limited availability of testing kits early in the pandemic. Approximately one in ten of these cases resulted in presentation or admission to hospital (7/66; 10.6%), and there was no mortality reported due to COVID-19. These findings are consistent with recently published data, which shows SARS-CoV antibody seroprevalence in blood donors in London to be 17.5% in week 18 of 2020 [11].

The potential effect of COVID-19 infection on patients with adrenal insufficiency could be partially mitigated if appropriate sick day management is followed. At the start of the lockdown resources were provided to relevant patients outlining steroid emergency rules. These patients were also reminded that they were in the UK Government’s ‘clinically vulnerable’ group and that they should follow stringent social distancing precautions. Patients who had not previously attended a steroid education session were educated via the telephone during lockdown and were mailed an emergency intramuscular hydrocortisone injection. In the group surveyed, patients prescribed regular long-term replacement steroids were well aware of steroid emergency rules. Fifty nine patients (59/159, 37.1%) of this group had taken part in a telephone education session offered to them by our endocrine department after lockdown had commenced. This was one of the Society for Endocrinology and UCLH endocrine departmental priorities to ensure patient safety in secondary adrenal insufficiency during the pandemic [4]. This indicates that despite the unexpected and unprecedented nature of the COVID-19 pandemic, the majority of patients already possessed the knowledge to manage their own care in regards to steroid emergency cover.

In our centre, very few consultations were undertaken by telemedicine prior to 2020. During the lockdown, hospital resources were redirected towards acute medical care. Nationwide patients were asked to present to the hospital only in an emergency. From the 23rd of March, the majority of the pituitary service outpatient appointments were conducted via the telephone rather than face to face. A small percentage of outpatient appointments were cancelled completely due to staffing shortages. Emergency face-to-face consultations were reserved for a very limited group of patients. Imaging and pathology planned as part of routine care were cancelled, only performed when deemed absolutely necessary. Patients were notified of these changes or cancellations via mobile phone text messages and/or letters in the post.

Consequently, 267 (64.8%) patients surveyed had a change or delay to the planned care for their pituitary disease. The following three areas are time critical in the management of patients with pituitary disease: 1) visual compromise, 2) suspected cancer and 3) the effects of a functioning tumour [1]. We did not identify any patients in these groups (known to our service prior to the pandemic) who have had adverse visual outcomes or a delay in cancer diagnosis and management from a delay in care. It is more difficult to assesses the possible longer-term adverse effects of tumour regrowth and over-secretion of pituitary hormones from delays in investigation and management with surgery, radiotherapy or medical treatment. Two patients experienced a delay in surgery, four experienced a delay in radiotherapy and five experienced a delay in taking medication to suppress pituitary hormone overproduction (one cabergoline and four lanreotide). Whilst these numbers are small, it is possible that different treatment decisions could have been made for some patients, were face to face examination and up to date pathology or imaging available during lockdown.

This survey assessed change or delay to planned care over a 14-week period (from the start of lockdown on the 23rd March until the date the patient was called between the 23rd May to the 30th June). Patients with stable pituitary disease are routinely reviewed by our service every six months, so some patients would not have had a review or investigations planned in this period anyway.

Whilst some of our staff were redeployed to work in other areas of the hospital, in our department 50% of medical and 40% nursing staff were able to continue to conduct telephone consultations and answer patient queries via email or telephone. This continuation of support may help to explain why patients, despite experiencing delays to services, remained relatively positive when asked about the impact that the pandemic has on their care during the period. Although not formally assessed in the survey, many patients expressed to the interviewer they understood the need for NHS services to be prioritised during this period and that they accepted it was necessary to manage the risk of contracting the virus by avoiding attending the hospital for routine appointments or investigations.

Given we are only a handful of months into the pandemic, research into the effects of the COVID-19 pandemic on patient care are not widely reported. Data recently published by the NHS has shown the large scale of cancelled elective surgeries and a sharp rise in the proportion of cancer patients who have experienced delays in assessment and/or treatment during April 2020 [12, 13]. Health care modelling has shown that modest delays in surgery for cancer incur significant impact on survival [14]. Now as the lockdown eases and non-urgent care for patients with chronic medical conditions resumes, further research into the scale and potential impact of such delays will be required in all fields, including endocrinology.

Numerous patients spontaneously reported in the general comments section of the survey, the negative impact of the COVID-19 pandemic on their mental health. The negative psychological effects of quarantine have been recognised under other circumstances and is an area that requires ongoing research related to this current global pandemic [15, 16].

### Limitations

A limitation of this study was that 28% of patients identified were unable to complete the survey as they had not been contactable via phone after two attempts. It is possible that this group includes patients who were uncontactable due to death, severe illness or hospitalisation. We did, however, attempt to mitigate this by reviewing the relevant records of patients that were not contactable. Moreover, the fact the proportion of patients we surveyed with suspected or confirmed COVID-19 was similar to that in the local population (16% versus 17.5% respectively) is reassuring [11].

Our institution is the largest pituitary centre in the United Kingdom, thus the findings of this survey may not be representative of smaller endocrinology services who may have had fewer staff available for specialist service provision during the pandemic with all focus on acute services.

### Conclusions

A significant number of patients with pituitary disease have experienced a delay in their care. Identifying patients with pituitary disease who have been impacted by COVID-19 allows prioritisation of services and resources as the pandemic continues. Focussing the resources on patient education to enable efficient self-management in secondary adrenal insufficiency is an effective use of limited resources. To ensure that lessons are learnt from experiences during this pandemic, further research is required to identify and address areas of improvable care standards, as well as to embed examples of success and good practice.

## Data Availability

The datasets generated and analysed during the current study are available from the corresponding author on reasonable request.
